# An Indian perspective on primary angle closure and glaucoma

**DOI:** 10.4103/0301-4738.73687

**Published:** 2011-01

**Authors:** Ramanjit Sihota

**Affiliations:** Dr. R. P. Centre for Ophthalmic Sciences, All India Institute of Medical Sciences (AIIMS), Ansari Nagar, New Delhi - 110 029, India

**Keywords:** Primary angle closure, glaucoma, India, primary angle closure glaucoma, primary angle closure disease

## Abstract

**Aim:**

To provide a synopsis of primary angle closure disease in India, and Indian studies on the same.

**Results:**

Primary angle closure glaucoma forms almost half of all adult primary glaucomas seen in a hospital setting in India. Anatomically, corneal diameters and anterior chamber depths were least in acute and chronic PACG eyes as compared to subacute eyes and controls. Besides relative pupillary block, a Valsalva maneuver during activities of daily living may be responsible for intermittent angle closure and raised IOP in predisposed eyes. Iridotomy alone, controlled the intraocular pressure in 66.7% of subacute eyes and 12.9% of the acute. Medical therapy was additionally required for 35.5% of the acute eyes, 12.1% of the subacute and 30.0% of the chronic cases. There was a greater mean and peak IOP reduction, achieved with 0.005% latanoprost once daily, 8.2 ± 2.0 mm Hg, compared with 0.5% timolol twice daily, 6.1 ± 1.7 mm Hg2. A progression of PACS to PAC was seen in 22%, PAC to PAC OHT in 38.7% and PAC OHT to PACG in 30.7% over 5 years.

**Conclusions:**

Primary angle closure disease is common in India, and can be managed well with iridotomy, followed by an appropriate control of IOP.

Primary angle closure glaucoma (PACG) is a protean disease with a differing incidence, indeterminate initial stages and a varied presentation in different races. The term angle closure glaucoma evokes diverging images for different ophthalmologists, and there are many classifications with subgroups overlapping each other. Primary angle closure (PAC) has been studied carefully over the last decade, but there is much more that needs to be done.

There is a significantly high incidence of PACG in India, which forms almost half of all adult primary glaucomas seen in a hospital setting.[1,2] In Indian population-based studies, the prevalence of PACG varies in different surveys due to methodology and age of the patients studied, as well as other parts of the world [Tables 1 and 2].[3–9]

**Table 1:** Details of various population-based studies carried out in India

Study	Age group (years)	PACS	PAC	PACG
VES (1998)	30–60			43.2
APEDS (2000)	>30	1.41%		0.71%
West Bengal Rural	>50			0.23%
Study (2005)				
ACES rural (2003)				0.5%
CGS – Rural (2006)	>40	6.27%	0.71%	0.87%
CGS: Urban	>40	7.24%	2.75%	0.88%

VES: Vellore eye survey, APEDS: Andhra Pradesh eye disease survey, ACES: Aravind comprehensive eye survey, CGS: Chennai glaucoma study, PACS: Primary angle closure suspect, PAC: Primary angle closure, PACG: Primary angle closure glaucoma

**Table 2:** Prevalence of primary angle closure glaucoma around the world

Race/location	Prevalence of angle closure glaucoma in older age population (generally over age 40)
White populations (Europe, Australia)	0.1–0.6%
Asian populations (Alaska, Japan,	0.3–2.7%
Mongolia, Singapore, India)	
Hispanic population (US)	0.1%
Black populations (Africa)	0.5–0.6%

The clinical spectrum of PAC and its glaucoma as used here are[10,11]:

- Subacute or intermittent PAC is intermittent, self-limited, intraocular pressure (IOP) elevations accompanied by prodromal symptoms of headache, haloes and blurred vision, but with normal IOP in the interparoxysmal period, in patients with an occludable angle.
- Acute PACG: Such eyes have IOP-induced corneal edema (experienced as blurred vision and multicolored halos around lights), vascular congestion and pain. This may be accompanied by nausea and vomiting. Patients also have congestion of the eye, corneal edema, a vertically mid dilated pupil and very high IOPs.
- Chronic PACG: Patients with occludable angles having peripheral anterior synechiae of more than 180 degrees and a chronically elevated IOP. Some patients have no symptoms, “creeping PACG,” whereas others complain of headaches occasionally.

## Indian Studies on Pathogenesis

### Structural factors

Acute PACG eyes were mildly hyperopic. All PAC subgroups had similarly short eyeballs and a steeper corneal curvature as compared with the control eyes.[10,11] Acute PACG lenses were thicker than all other PAC subgroups, but all PACG eyes had thicker lenses than controls. Corneal diameters and anterior chamber depths were least in acute and chronic PACG eyes as compared with subacute eyes and controls.[10,11] The uninvolved fellow eyes in each subgroup differed from the affected eyes of the same individual, only, in having more posteriorly positioned lenses. Biometric differences may explain, to some degree, the disparate presentations of the subtypes of PACG.[1,2] On ultrasound biomicroscopy, eyes with PACG had a thinner iris, with a shorter trabecular iris angle, angle opening distance and trabecular ciliary process distance.[12] Eyes with acute PACG had the narrowest angle recess.[1,2]

In a study from South India, a shorter axial length was seen in an occludable angle group 22.07 ± 0.69 mm as compared with normal eyes (22.76 ± 0.78 mm).[13] The anterior chamber depth was shallower in eyes with occludable angles (2.53 ± 0.26 mm) compared with normal subjects (3.00 ± 0.30 mm), and the lens thickness was greater in people with occludable angles (4.40 ± 0.53 mm) compared with 4.31 ± 0.31 mm in normal subjects. Table 3 shows the ocular parameters in the subtypes of PAC and control eyes and Table 4 shows the comparison of various studies of acute PACG eyes in Indians and in other races.

**Table 3:** Ocular parameters in the subtypes of PAC and control eyes

	I Acute PACG eyes (*n* = 30)	II Chronic PACG eyes (*n* = 29)	III Subacute PAC eyes (*n* = 30)	IV Controls (*n* = 30)
Age (years)	55.77 ± 6.6	54.07 ± 8.9	50.87 ± 7.2	54.4 ± 8.1
Refractive error (diopters)	+ 1.962 ± 0.81	+ 1.013 ± 0.71	+0.958 ± 0.72	+1.219 ± 0.55
Corneal diameter(mm):				
Horizontal	11.07 ± 0.21	11.26 ± 0.25	11.53 ± 0.34	11.68 ± 0.31
Vertical	10.70 ± 0.25	10.79 ± 0.25	11.12 ± 0.28	11.23 ± 0.25
Keratometry:				
Central	45.33 ± 1.72	44.50 ± 1.54	44.90 ± 1.34	43.76 ± 0.79
Peripheral	45.10 ± 1.84	44.20 ± 1.39	44.67 ± 1.56	44.02 ± 0.90
Pachymetry:				
Central	572.7 ± 35.6	524.2 ± 30.8	531.4 ± 25.3	524.5 ± 12.8
Peripheral	689.0 ± 40.5	668.8 ± 53.58	691.7 ± 46.7	696.3 ± 13.6
Corrected AC depth (mm)	1.533 ± 0.10	1.863 ± 0.17	2.173 ± 0.33	2.329 ± 0.17
Lens thickness (mm)	4.902 ± 0.19	4.619 ± 0.30	4.600 ± 0.31	4.430 ± 0.13
Axial length (mm)	22.56 ± 0.63	22.61 ± 0.88	22.88 ± 0.88	23.49 ± 0.86
Relative lens position	0.202 ± 0.008	0.208 ± 0.011	0.219 ± 0.014	0.216 ± 0.010

PAC: Primary angle closure, PACG: Primary angle closure glaucoma, AC: Anterior chamber

**Table 4:** Comparison of various studies of acute PACG eyes in Indians and in other races

	Sihotai[11]	Lowe[33]	Sood[10]	Wilenskyi[34]	Tomlinson *et al*.[35]	Zhao *et al*.[36]	Markowitz[37]	Sakai *et al*.[38]
Refractive error	+ 1.962 ± 0.81			2.25	±1.09			
Corneal diameter	10.88		10.89		10.72			
Keratometry	7.46	7.61	7.41		7.55			
Pachymetry	0.573 ± 0.835	0.533	0.64	0.532	0.563			
“True” AC depth	1.53 ± 0.10	1.8	1.8 ± 0.25	1.74		1.88 ± 0.20		1.9 ± 0.41
Lens thickness	4.90 ± 0.19	5.9	5.18	5.34	5.23		4.99	
Axial length	22.56 ± 0.63	22.01	23.09	21.86	22.06		21.62	
Relative lens position	0.202	0.20		0.224				

PACG: Primary angle closure glaucoma, AC: Anterior chamber

### Pathophysiology

Besides relative pupillary block, there are many other physiological changes that result in PAC, e.g. dilation in a prominent last roll of the iris or plateau iris configuration. The occurrence of a Valsalva maneuver or similar physiological changes during activities of daily living may be responsible for intermittent angle closure in predisposed eyes as well as large, acute elevations of IOP, which could be detrimental for a glaucomatous optic neuropathy.[14]

## PAC and Glaucoma Presentation in India

In a study in a North Indian hospital,[2] PAC and glaucoma constituted 45.9% of all primary adult glaucomas seen. 24.8% of these patients were diagnosed as having acute angle closure glaucoma, 31.2% subacute PAC and 44% a chronic PACG. Patients with PAC and glaucoma showed a male:female ratio of 48.6:51.4. Females predominated in the acute subgroup (79.8%) and in the subacute group (66.7%). Males were more commonly affected in the chronic subtype, 75.4%. PAC and glaucoma occurred between 30 and 80 years, with the maximally affected decade being the sixth. Acute PACG predominated in the third and fourth decade and subacute PAC between the fourth and fifth decades of life. Above 60, the distribution of the subtypes was almost equal.

Colored haloes were seen most often by patients having an acute attack, i.e. 64.5%. Only 35.3% of those having subacute attacks complained of haloes and only 18.2% of the patients having chronic PAC complained of the same. Ocular pain was most frequent in acute and subacute eyes, 62.1% and 45.5%, respectively. Associated vomiting occurred in 35.5% of the acute cases and in 19.9% of the subacute PAC patients, and was negligible in chronic PAC. A nonspecific headache and a diminution of vision were commonly encountered in all subtypes of glaucoma. More than 80% of the chronic PACG eyes had no significant symptoms.

Bilaterally affected eyes were seen in 95.5% of subacute PAC, 64.1% of chronic PACG and only 35.5% of acute PACG. An absolute eye was most often seen with chronic angle closure glaucoma (32.3%) and following an acute attack (15.3%). Seven percent were bilaterally blind. Only 8.9% of the patients who had had an acute PACG achieved a final vision of 20/40 or better.

## Clinical Features

Gonioscopy is an essential component of the diagnosis of PAC. However, in India, the facilities or expertise may not always be available and, thus, easier alternatives or corroborative means are necessary. The sensitivity and specificity on the flashlight test were 45.5% and 82.7%, respectively.[15] For the van Herick’s test, they were 61.9% and 89.3%.[15] The flashlight test and van Herick’s test are therefore of limited use individually as screening tests for occludable angles.[2]

**Figure 1:**
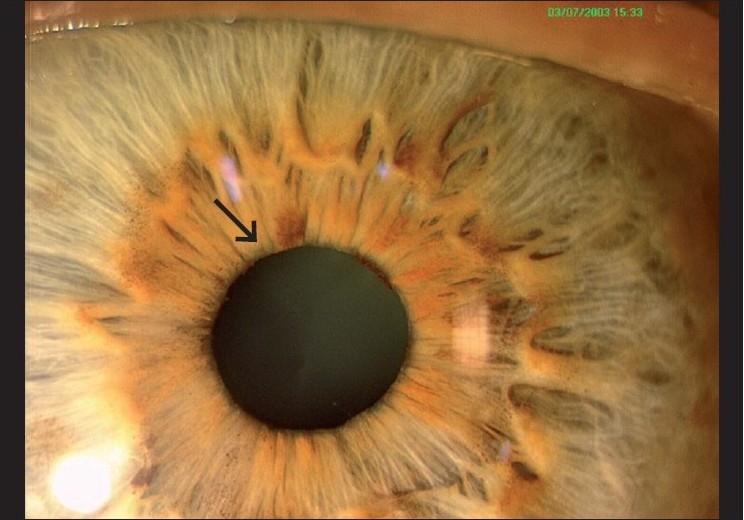
Pupillary ruff atrophy (The white arrow shows atrophy of pupillary ruff while the arrow shows normal anatomy)

Pupillary ruff atrophy is easily seen with a slit lamp or even a torch as a loss of a part or whole of the pigmented frill around the pupil. No primary open angle glaucoma (POAG) eye had an abnormal pupillary ruff. A total of 86.7% of the subacute PACG eyes and all eyes with acute and chronic PACG showed some grade of pupillary ruff atrophy.[16] A combination of anterior chamber depth assessment and pupillary ruff evaluation [Fig. 1] may highlight angle closure when a good gonioscopy is not possible. Seventy percent of eyes having a form of PAC showed a positive dark room prone provocative test (DRPPT), which can also be performed by any ophthalmologist with any tonometer.[16]

Subacute PAC was commonly seen between 40 and 59 years. The gonioscopic picture was that of an occludable angle, apparently closed in areas, which, after YAG iridotomy, opened in two-thirds of such eyes. A third showed a persistent rise of IOP after the iridotomy.[1,2]

Acute PACG was common in the age group 50–59 years. After controlling the initial high IOP medically, an iridotomy alone was able to control the IOP in 12.9%, but a filtering surgery was required ultimately in 51.6%. The final visual acuity was <20/40 in 91.1% of the eyes.[1,2]

**Figure 2:**
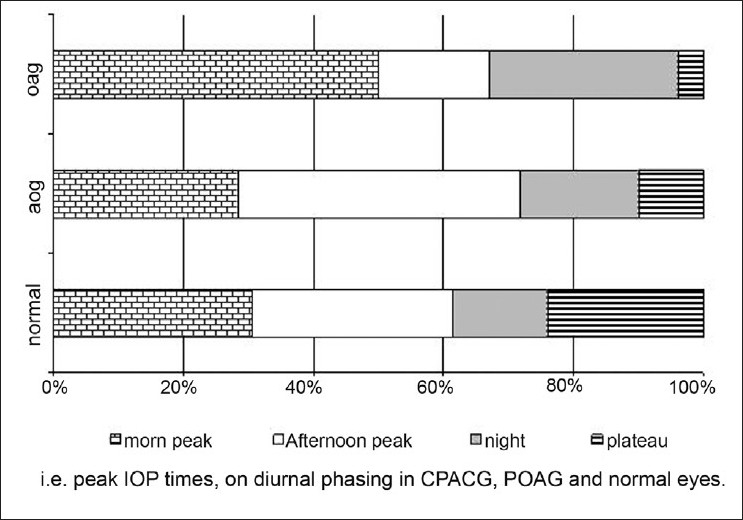
Occurrence of different patterns of diurnal curves

Eighty percent of the chronic PACG eyes were asymptomatic. Diurnal pressure fluctuations were significantly higher in the chronic PACG, 7.69 ± 3.03 mmHg, and POAG, 8.31 ± 2.58 mmHg, eyes compared with controls, 4.83 ± 2.46. At 7 and 10 am, IOPs peaked more often in POAG eyes as compared with chronic PACG eyes; however, afternoon peaks were more common in chronic PACG eyes.[2,17–19] Fig. 2 depicts the proportionate occurrence of different patterns of diurnal curves, i.e. peak IOP times, on diurnal phasing in chronic PACG, POAG and normal eyes. None of the eyes had a controlled IOP after iridotomy; a third were controlled on topical medication and the rest required surgery with good results in the short term. Fifteen percent had characteristic glaucomatous field defects and 13.2% had an advanced visual field defect.

Iridotomy alone controlled the IOP in 66.7% of the subacute eyes and in 12.9% of the acute eyes. Medical therapy was additionally required for 35.5% of the acute eyes, 12.1% of the subacute eyes and 30.0% of the chronic cases.[2] Nd YAG laser peripheral iridotomy (LPI) alone or with topical medication controlled the IOP in 48.3% of the acute ACG, 78.8% of the subacute eyes and 30% of the chronic eyes.[2]

Medications in PACG are the same as those used in POAG eyes.[20–24] There are greater mean and peak IOP reductions achieved with 0.005% latanoprost once daily, 8.2 ± 2.0 mmHg, compared with 0.5% timolol twice daily, 6.1 ± 1.7 mmHg.[21] Bimatoprost 0.03% monotherapy improved ocular blood flow and provided a better diurnal IOP control than concomitant timolol–pilocarpine in eyes with chronic PACG, and was well tolerated.[23,24] Trabeculectomies have been performed with a good success for the control of IOP, with few long-term problems.[25,26]

## Progression in PAC

In a population-based study of primary angle closure suspect (PACS) the 5-year incidence of PAC was 22%, as reported by Thomas *et al*.[27] A hospital-based study on the course of PACS subjects after LPI found that 28% progressed to PAC. Decreasing anterior chamber angle (ACA) was the predictive factor for the progression of PACS to PAC.[28] There was no increase in IOP, history or symptoms of acute attack of glaucoma among the study subjects after LPI.[2] Another hospital-based study reported that after LPI, no eye with PACS progressed to PAC or PACG.[29]

Progression of PAC to chronic PACG is characterized by glaucomatous optic neuropathy and chronically raised IOP. Thomas *et al*. reported that 28.5% of the PAC subjects had progressed to PACG; two of seven with appositional and six of 21 with synechial closure over 5 years in a population study.[30] In a study over 5 years, a third of PAC eyes with very narrow angles, higher global indices, intervisit IOP fluctuations and a longer duration of follow-up were observed to develop ocular hypertension. 30.7% of these PAC eyes with ocular hypertension developed a glaucomatous visual field defect.[31] That is, 11.1% of PAC or subacute PAC eyes in a hospital-based Indian study developed glaucomatous visual field defects or chronic/primary ACG over 5 years. All these eyes had been previously noted to have a chronically raised IOP and were on glaucoma therapy thus highlighting the significance of a chronically raised IOP in eyes that had earlier been recorded to have a normal diurnal phasing. Table 5 shows the baseline parameters among perimetrically stable PAC eyes with normal IOP, PAC with hypertension having stable fields and those that developed a visual field defect, i.e. PACG on follow-up.

**Table 5:** Baseline parameters among perimetrically stable PAC eyes with normal IOP, PAC with hypertension having stable fields and those that developed a visual field defect, i.e. PACG on follow-up

Variable	Stable PAC eyes with normal IOP (*n* = 46)	Stable PAC eyes with chronically raised IOP (*n* = 18)	PACG eyes (*n* = 8)	One-way ANOVA^*^
Age (years)	48.5 ± 8.7	59.2 ± 8.6	54.9 ± 9.2^*^	**<0.001**
Follow-up (years)	4.7 ± 0.8	6.2 ± 2.1	6.89 ± 2.4^*^	**0.01**
Cup:disc ratio	0.53 ± 1.5	0.59 ± 0.1	0.62 ± 0.1	0.1
Central corneal thickness (microns)	552 ± 30.7	541 ± 39.1	540 ± 35.6	0.6
Angle recess (degrees)				
>10	36 (78%)	4 (22%)	1 (12.5%)^*^	**0.01**
≤10	10 (22%)	14 (78%)	7 (87.5%)	
Extent of PAS (quadrant)^**^				
<1	28 (61%)	5 (28%)	3 (37.5%)	0.5
1–2	18 (39%)	8 (72%)	5 (62.5%)	
Phasing – IOP max (mmHg)	20.1 ± 4.3	21.5 ± 4.5	20.6 ± 1.1	0.06
Phasing – IOP min (mmHg)	14.1 ± 3.3	15.1 ± 3.6	15.2 ± 1.6	0.09
Phasing difference	4.9 ± 1.5	6.3 ± 3.9	5.8 ± 1.6	0.07
DRPPT min (mmHg)	15.7 ± 3.5	15.7 ± 4	16.5 ± 1.5	0.9
DRPPT max (mmHg)	20.5 ± 4.1	20.2 ± 4.3	21.5 ± 6.3	0.8

PACG: Primary angle closure, PACG: PAC glaucoma, IOP: Intraocular pressure, PAS: Peripheral anterior synechiae, DRPPT: Dark room prone provocative test, Bold numbers show a significant *P* value

Thomas *et al*.[30] re-examined 37 patients diagnosed as PAC during a population-based study, after 5 years, during which no therapy was given. A progression to PACG was noted in 28.5% based on optic disc damage and field defects on automated perimetry. One of nine patients, i.e. 11.1%, who underwent iridotomy progressed, as compared with seven of 19, 36.8%, which had refused an iridotomy. They could not identify any features that predicted progression, but showed that an iridotomy reduced the progression to glaucomatous optic neuropathy. A hospital-based study[31] noted that 9.3% of eyes with PAC progressed to PACG. Patients with ≤2 quadrants of angle closure at baseline had 7.7% odds of progression compared with 100% odds in patients with >2 quadrants of angle closure (risk ratio 12.9).

Acute angle closure is usually treated by immediately lowering the IOP. However, many patients will go on to develop chronic PACG if the acute attack lasts for more than 1 week, although this occurs more frequently in Asians than in Caucasians. Table 1 shows the incidence of chronic raised IOP, glaucomatous optic neuropathy and blindness in the different ethnic groups. Table 6 shows progression in acute angle closure patients.

**Table 6:** Progression in acute angle closure patients

	Singapore %	India %	Caucasians %
Chronically raised intraocular pressure	50.0	71.0	35.0
Glaucomatous optic neuropathy	50.0	39.0	12.5
Blindness	17.8	15.3	Nil

About 15% of the chronic PACG eyes were found to progress over 5 years, especially those that started with larger cup:disc ratios, and a higher deviation of MD and PSD values on Humphrey visual fields.[31] They also had a higher IOP, 18.07 ± 7.35 mmHg, at 5 years. Patients who underwent surgery had a significantly greater chance of progression of both disc and field parameters when re-examined at 5 years.

A study of the long-term prognosis for chronic ACG found that 79% of the patients achieved IOP control and stable visual fields, with only 35% requiring trabeculectomy over a 5-year follow-up.[31] Table 7 shows the comparison of chronic PACG eyes showing visual field progression and those with stable fields.

**Table 7:** Comparison of chronic primary angle closure glaucoma eyes showing visual field progression and those with stable fields

	Stable visual fields (*n* = 47)	Progressive visual fields (*n* = 13)	*P*-value
Age at presentation (years)	57.95 ± 9.14	58.76 ± 9.41	0.78
HT/DM			
Visual acuity			
Baseline	0.67 ± 0.24	0.66 ± 0.20	0.90
5 years	0.68 ± 0.25	0.64 ± 0.25	0.63
Cup:disc ratio			
Baseline	0.56 ± 0.21	0.64 v 0.16	0.18
5 years	0.58 ± 0.21	0.71 ± 0.17	0.06
Intraocular pressure			
Baseline	20.4 ± 4.9	20.8 ± 5.15	0.79
5 years	15.0 ± 3.33	18.07 ± 7.35	0.17
MD			
Baseline	-9.48 ± 7.99	-14.84 ± 9.59	**0.04**
5 years	-8.88 ± 8.36	-17.58 ± 9.90	**0.002**
PSD			
Baseline	6.25 ± 3.49	7.08 ± 2.75	0.45
5 years	6.09 ± 3.74	8.45 ± 3.54	0.048

Bold numbers show a significant *P* value

Chronic PACG eyes appeared to progress faster and to a greater extent on perimetry, and were more resistant to changes in optic nerve head topography following therapy, requiring a larger percentage drop in IOP to manifest improved optic nerve head parameters, as compared with the POAG eyes.[31]

Fellow eyes of patients with acute angle closure are likely to develop angle closure. A study in India found that 22% of the fellow eyes developed evidence of angle closure or peripheral anterior synechiae following iridotomy, although none developed chronically raised IOP within 5 years.[32] However, 50% of the fellow eyes that did not have an iridotomy progressed to acute ACG.[33]

In summary, in India, all eyes that show an anatomical or familial predisposition for PAC should have an iridotomy. Patients showing evidence of PAC, i.e. an occludable angle with peripheral anterior synechiae, with or without a raised IOP, should also have an iridotomy, after controlling any raised IOP. Acute PACG eyes present late and frequently require a trabeculectomy to control IOP, but visual prognosis is generally poor. Chronic PACG eyes appear to progress faster than POAG eyes. This may be due to continuing periodic angle closure and raised IOP and due to pathomechanisms other than relative pupillary block. Therefore, medications should be re-evaluated frequently and trabeculectomy may be required if perimetric progression is seen on topical medication. The success rate of trabeculectomies in Indian chronic PACG eyes is good, with few complications.
